# A Novel Mutation in a Patient with Wiskott-Aldrich Syndrome

**DOI:** 10.4274/tjh.galenos.2020.2019.0321

**Published:** 2020-05-06

**Authors:** Yurday Öncül, Arzu Akyay, İbrahim Tekedereli

**Affiliations:** 1 İnönü University Faculty of Medicine, Division of Pediatric Hematology, Malatya, Turkey; 2 İnönü University Faculty of Medicine, Division of Medical Genetics, Malatya, Turkey

**Keywords:** Wiskott-Aldrich Syndrome, WAS gene, Novel mutation

## To the Editor,

We read with great interest the recently published article in your journal by Kaya et al. [1] regarding a novel mutation in the Wiskott-Aldrich syndrome (*WAS*) gene. After that publication, we also had a patient with another novel mutation in the *WAS* gene from Turkey.

**Figure 1:**
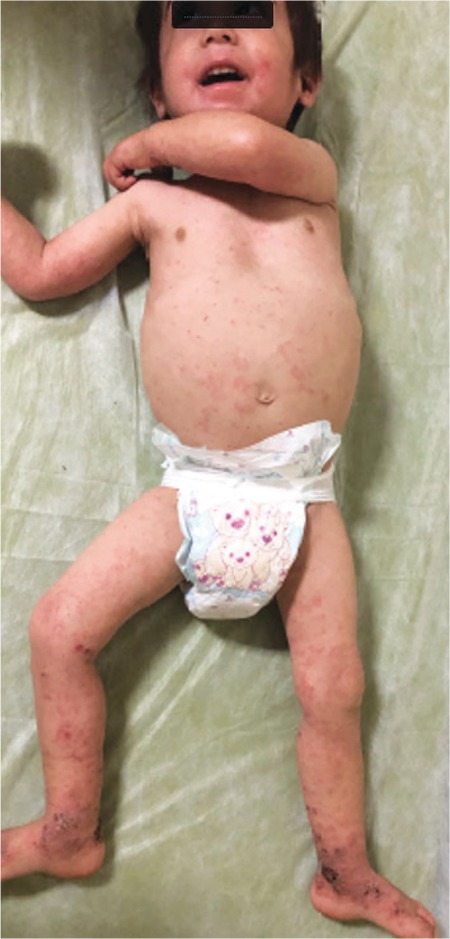
Physical examination revealed diffuse eczema, widespread petechiae, and pulmonary crepitant rales and rhonchi.

**Figure 2:**
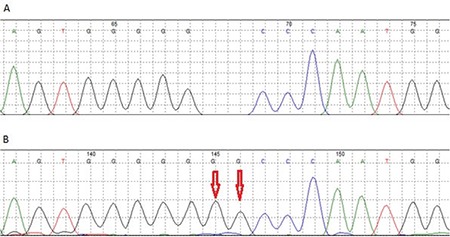
Hemizygous c.11_12insGG p.G4Afs mutation on exon 1 (A, B).

A 3-month-old boy was admitted to our hospital with the complaints of cough, wheezing, and eczema. He also had a history of pneumonia. On physical examination, diffuse eczema was observed (Figure 1), along with widespread petechiae and pulmonary crepitant rales and rhonchi. His family history was unremarkable. Laboratory analysis revealed anemia (hemoglobin of 8.9 g/dL), leukocytosis (white blood count of 13,330/mm^3^), andthrombocytopenia (platelet count of 63,000/mm^3^). Mean platelet volume was 4.8 fL. A peripheral blood smear revealed thrombocytopenia and micro-thrombocytes. Immunoglobulin levels were normal. Peripheral lymphocyte subset analysis revealed reduced CD3 percentage and CD16/CD56 ratio. With these results, patient was diagnosed with WAS, and molecular genetic analysis revealed a novel mutation in the *WAS* gene, a hemizygous c.11_12insGG p.G4Afs mutation on exon 1 (Figure 2). The patient is 18 months old now. Human intravenous immunoglobulin therapy was administered monthly, and thrombocyte replacement was done in case of need [2]. He did not have a family donor, so he was scheduled for allogeneic hematopoietic stem cell transplantation from an unrelated donor.

The clinical presentation of WAS is very heterogeneous. Based on the severity of symptoms, a 5-point severity score was developed [2,3]. This score differentiates patients with milder presentation (scores of up to 2) from the severe classic WAS phenotype (scores of 3-5). While most patients suffer from thrombocytopenia and susceptibility to infections, the other clinical complications of the disease can be variably present [4]. WAS cases with milder clinical manifestations are usually referred to as X-linked thrombocytopenia (XLT) [4,5]. XLT patients must also be carefully monitored, however, because patients with initially mild phenotypes can transition to severe phenotypes. Our patient presented a classical severe case of WAS with severe eczema, immune deficiency with recurrent infections, and thrombocytopenia.

There are many possible mutations in the *WAS* gene. However, missense mutations are seen most often, especially on the first four exons. The subsequently most frequent mutations are splice mutations, deletions, insertions, nonsense mutations, and complex mutations in descending order [3,4]. The missense mutations on exons 2 and 3 are linked to mild phenotypes [5]. The nonsense mutations, insertions, and deletions are often associated with severe phenotypes. In our case, a hemizygous c.11_12insGG p.G4Afs mutation on exon 1 was detected. This was a frameshift mutation. Frameshift mutations encode incorrect amino acids and usually cause nonsense codons [6]. Another frameshift mutation at the c:11 position on the *WAS* gene with deletion of a guanine nucleotide has been reported as a disease-causing variant [7]. In our case, an insertion affected the WAS protein and led to the emergence of the disease. Therefore, the 11^th^ position on *WAS* cDNA could be referred to as a mutational hotspot. To the best of our knowledge, this is the first case of this mutation to be presented. We suspect that this mutation might be important in contributing to the genotype-phenotype relation.
